# Traditional Chinese Medicine for adolescent idiopathic scoliosis: a systematic review and meta-analysis

**DOI:** 10.3389/fped.2025.1502741

**Published:** 2025-09-01

**Authors:** Peng Li, Yue He, Jihe Zhou, Min He

**Affiliations:** ^1^ School of Physical Education and Health, Zunyi Medical University, Zunyi, China; ^2^ Sichuan Provincial Ba-Yi Rehabilitation Center, And Affiliated Rehabilitation Hospital of Chengdu University of Traditional Chinese Medicine, Chengdu, Sichuan, China; ^3^ Chengdu Sports University, Chengdu, China; ^4^ Longjing Middle School, Renhuai, Guizhou, China

**Keywords:** Traditional Chinese Medicine (TCM), adolescent scoliosis, non-surgical treatment, spinal deformity, complementary therapy

## Abstract

**Objective:**

To evaluate the clinical effectiveness of Traditional Chinese Medicine (TCM) interventions—such as spinal manipulation, Daoyin exercises, acupuncture, and massage—in adolescents with idiopathic scoliosis, and to summarize structured treatment regimens for clinical reference.

**Methods:**

A systematic search was conducted in PubMed, Embase, Web of Science, CNKI, VIP, and Wanfang databases from inception to March 2024. Randomized controlled trials (RCTs) comparing TCM with conventional treatments in adolescent idiopathic scoliosis were included.

**Results:**

TCM interventions—ranging from spinal manipulation and Daoyin corrective exercises to acupuncture and massage—were delivered 2–5 times per week over 4–12 weeks. Meta-analysis showed a significant reduction in Cobb angle (MD = −3.97, 95% CI: −4.24 to −3.71, *p* < 0.00001) and increased total effectiveness (OR = 4.42, 95% CI: 3.22–6.08, *p* < 0.0001). Egger's test and funnel plots showed no major publication bias. Subgroup analysis indicated consistent outcomes across different TCM techniques.

**Conclusion:**

This meta-analysis demonstrates that TCM interventions—particularly spinal manipulation, Daoyin exercises, acupuncture, and massage—are effective in improving both structural and clinical outcomes in AIS. A practical regimen of manual therapy (2–3 times/week), daily Daoyin exercise, and supportive acupuncture and massage over 12 weeks offers a safe, non-invasive alternative to bracing or surgery, with high patient compliance.

**Systematic Review Registration:**

https://www.crd.york.ac.uk/PROSPERO/recorddashboard, identifier CRD42024589328.

## 1 Introduction

### 1.1 Background

Adolescent idiopathic scoliosis (AIS), affecting approximately 2%–3% of adolescents worldwide, is typically diagnosed during the adolescent growth spurt (1–3). AIS is characterized by lateral curvature with spinal rotation. If untreated, it may lead to functional impairment, pain, respiratory dysfunction, and reduced quality of life (4, 5).

### 1.2 Current treatment methods

Conventional treatments for AIS include bracing and surgery. Bracing is widely used during adolescence to slow spinal curvature progression. However, long-term bracing may negatively affect daily life and psychological wellbeing (6, 7). Manual therapy, including techniques like spinal mobilization, soft tissue manipulation, and postural correction, has gained recognition as a complementary treatment aiming to improve spinal alignment, reduce muscle tension, and enhance functional outcomes. Recent studies suggest that manual therapy can be a valuable non-invasive alternative or adjunct in the management of AIS (8). Surgery is typically reserved for severe cases. Though effective in correcting deformities, it carries risks of complications and long-term consequences (9, 10). Given the limitations associated with these conventional treatments, such as discomfort with bracing and risks associated with surgical intervention, exploring alternative non-invasive methods is increasingly important. Traditional Chinese Medicine (TCM), with its holistic and minimally invasive approaches, represents one such promising alternative.

### 1.3 The potential of Traditional Chinese Medicine (TCM)

In recent years, non-invasive Traditional Chinese Medicine (TCM) has been increasingly applied in spinal health care. According to TCM theory, scoliosis is related to disharmony of qi and blood and obstruction of meridians. TCM therapies such as acupuncture, tuina, and herbal medicine aim to regulate qi and blood, unblock meridians, relieve pain, and improve spinal function. Some clinical studies have shown that TCM treatments can have positive effects on adolescent idiopathic scoliosis patients, but systematic research evidence is still lacking to fully assess its efficacy (11–13).

### 1.4 Research objective

This systematic review and meta-analysis aim to evaluate the efficacy of TCM, particularly acupuncture and tuina, in improving Cobb angle, relieving pain, and enhancing quality of life in AIS. By integrating existing literature, this study seeks to systematically explore the impact of TCM treatments on AIS, providing scientific evidence for clinical practice and guiding future research directions.

## 2 Methods

This meta-analysis (CRD42024589328) was reported according to the Preferred Reporting Items for Systematic Reviews and Meta-Analyses (PRISMA) guidelines, ensuring transparency and completeness in the reporting process.

### 2.1 Information sources

A comprehensive search was conducted across three electronic databases (Web of Science, PubMed, Wangfang and EMBASE) for all articles published from the inception of each database up to August 20, 2024, with no language restrictions.

### 2.2 Eligibility criteria

Inclusion criteria:

This analysis included all randomized controlled trials (RCTs) investigating Traditional Chinese Medicine (TCM) therapies for the treatment of adolescent idiopathic scoliosis (AIS).

Interventions in experimental and control groups: studies were included if they compared:

1 a single TCM therapy with another treatment (e.g., tuina vs. exercise therapy);
2 TCM combined with another conservative treatment vs. the same conservative treatment alone (e.g., TCM + traction vs. traction alone);
3 TCM combined with a conservative treatment vs. a different conservative treatment combination (e.g., TCM + traction vs. traction + exercise).

Definition of AIS patients: Male or female adolescents aged 8–18 years with a spinal curvature of 10° or more as measured by the Cobb angle.

In short, studies were only included if the primary difference between the experimental and control groups was the use of TCM. Studies where TCM was compared to unrelated therapies (e.g., TCM + exercise vs. bracing) were excluded.

Primary outcome measures included overall treatment efficacy and changes in Cobb angle.

Exclusion criteria:

Non-randomized studies or studies with fewer than 3 participants in either the experimental or control group;

Studies in which the experimental treatment did not involve TCM;

Studies lacking outcome measures or without comparable results.

### 2.3 Literature search

A standard protocol for this search was developed, and controlled vocabulary was applied (as shown in Table 1).

**Table 1:** Search terms.

Search term classification	Term
Intervention method	(Zhong Yi Xue) OR (Traditional Chinese Medicine) OR (Acupuncture) OR (massage) OR (herbal medicine) OR (chiropractic manipulation) OR (Needle knife) OR (traction) OR (Acupuncture therapy) OR (Cupping therapy) OR (moxibustion) OR (massage for bone orthopedics) OR (Massotherapy) OR (Massage) OR (qigong) OR (Tai Ji) OR (Damo Channel-changing Scriptures) OR (baduan jin exercise)
Study subjects	(adolescent) OR (Adolescence) OR (Female Adolescents) OR (Male Adolescent) OR (Youth) OR (Teen) OR (Teenager)
Outcome indicator	"Cobb Angle” OR “Spinal Curvature"[Mesh] OR “Treatment Outcome"[Mesh] OR “Clinical Effectiveness” OR “Therapeutic Efficacy” OR “Quality of Life"[Mesh]

### 2.4 Data extraction

A standardized data extraction form was used to collect relevant information from each included study, including study design, sample characteristics, interventions, outcomes (e.g., Cobb angle, quality of life), and risk of bias (using the Cochrane tool). Two reviewers independently extracted the data and resolved discrepancies through discussion or consultation with a third reviewer. In cases of missing data, study authors were contacted; if no response was received, the study was excluded from quantitative synthesis but may have been included in qualitative analysis.

### 2.5 Literature quality assessment

**Figure 1:**
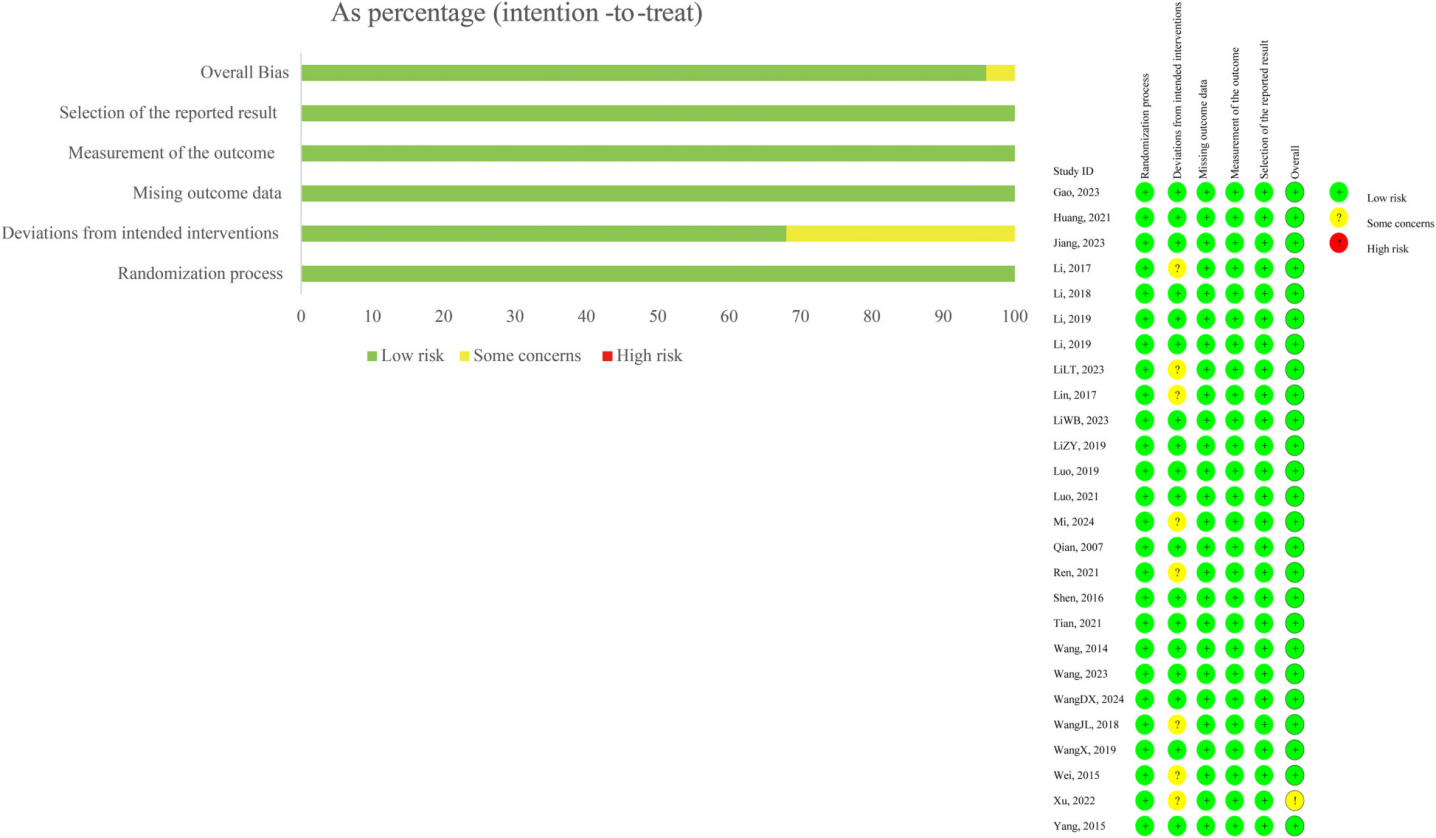
Literature quality assessment.

As shown in Figure 1: all 26 included studies were randomized controlled trials (RCTs), and their methodological quality was evaluated using the Cochrane Risk of Bias tool. Most studies clearly described the process of random sequence generation, but only a minority reported adequate allocation concealment. Due to the nature of TCM interventions such as manual therapy and Daoyin exercises, blinding of participants and personnel was generally not feasible, resulting in a high risk of performance bias. However, most studies adopted objective outcome measures (e.g., Cobb angle) assessed by independent examiners, reducing detection bias. Attrition bias was low overall, as dropout rates were minimal and comparable between groups. Reporting bias was considered low in most trials, although protocol registration was lacking in the majority. Overall, the included studies were of moderate methodological quality, and the main limitations stemmed from non-blinded designs and variability in intervention standardization.

## 3 Results

**Figure 2:**
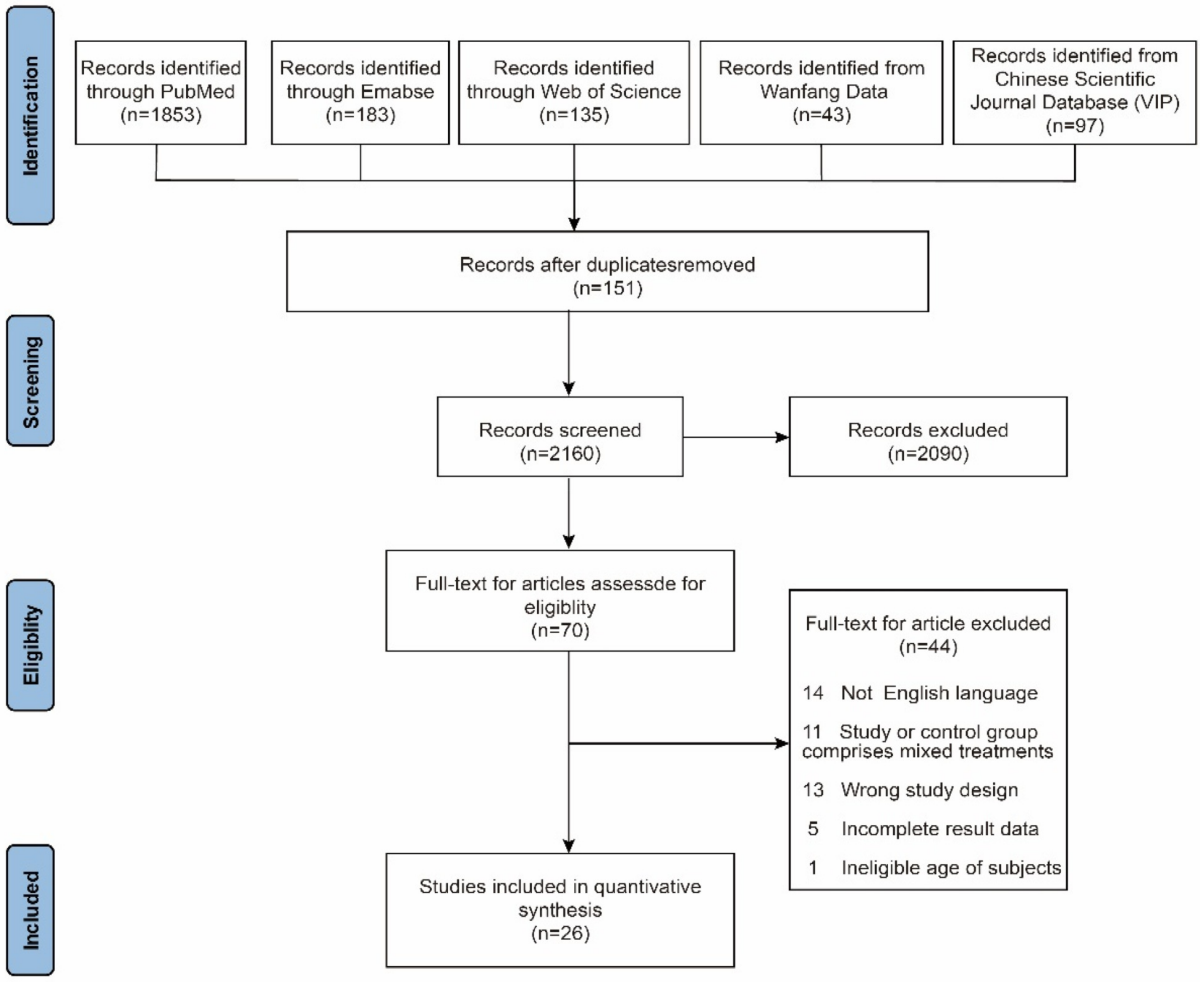
PRISMA flow diagram of study selection for the review on Traditional Chinese Medicine in adolescent scoliosis.

As shown in Figure 2: the initial database search identified 2,311 studies across multiple databases, with 1,853 articles from PubMed, 183 from Embase, 135 from Web of Science, 43 from Wanfang Data, and 97 from the Chinese Scientific Journal Database (VIP). After removing 151 duplicate entries, 2,160 records remained for screening based on titles and abstracts. Following this initial screening, 2,090 records were excluded for various reasons, such as irrelevant study focus, inappropriate population, or lacking necessary intervention details.

From the 70 full-text articles assessed for eligibility, 44 studies were further excluded due to various reasons: 14 studies were not in English, 11 studies contained mixed treatment groups (either in the intervention or control arms), 13 studies utilized an inappropriate study design (e.g., case studies or reviews), 5 studies had incomplete data, and 1 study involved participants outside the age range of interest. Finally, 26 studies met the inclusion criteria and were incorporated into the final quantitative synthesis, which evaluated the efficacy of Traditional Chinese Medicine (TCM) interventions for adolescent scoliosis, including changes in Cobb angle, pain reduction, and quality of life improvements. These TCM interventions encompassed treatments such as acupuncture, herbal medicine, and tuina.

Table 2 presents the basic characteristics of the included studies. A total of 26 randomized controlled trials were included, involving 2,031 adolescents with idiopathic scoliosis (1,055 in the intervention groups and 976 in the control groups), with ages primarily ranging from 8 to 18 years. The interventions included traditional Chinese manual therapies, spinal manipulation, acupuncture, three-dimensional traction, and Schroth exercises, with some studies incorporating herbal fumigation, Dao Yin techniques, and home-based rehabilitation. The treatment frequency typically ranged from 2 to 5 sessions per week, with durations from 10 days to 12 months.

**Table 2:** Summary of clinical studies on TCM manual therapy for scoliosis.

Study	Sample size (Exp./Ctrl.)	Age (Exp./Ctrl.)	Intervention method (Exp./Ctrl.)	Intervention duration (experimental group)	Intervention effects
Gao 2023	40/40	14.62 ± 0.68/15.73 ± 1.52	Tendon manipulation + TCM bone-setting/Orthotic brace + Functional exercise	2–3×/wk, 12 sessions/3mo	Cobb↓, rotation↓, mobility↑, 95% efficacy
Huang 2021	30/30	13.30 ± 2.50/12.93 ± 2.48	Acupuncture + traction + 3D spinal balance/Acupuncture + traction	1×/d, 2 courses (5wk)	Cobb↓, SRS-22↑, 96.7% efficacy
Jiang 2023	30/30	16 ± 2.16/15.5 ± 1.54	Long's bone-setting + traction + routine massage/Traction + routine massage	QOD (3wk)	Cobb↓, VAS↓, 86% efficacy
Li 2017	36/38	10–18/10–18	TCM spine manipulation + 3D traction + Boston brace/Boston brace	2–3×/wk manip + 1×/d tract (3mo)	Cobb↓, VAS↓, high efficacy
Li 2018	40/40	11.68 ± 1.69/12.23 ± 2.07	Relaxation + pelvic/lumbar/thoracic correction/Milwaukee brace	2×/wk (1wk break/2mo) + exer 20 min bid + counseling ×12mo	1. Cobb↓ (21.85°→10.83°); 2. AEMG↑ (1.53→1.11); 3. SDS/SAS↓ (*P* < 0.05)
Li 2019	40/40	8–16/8–16	Tendon manipulation + bone-setting/Brace + traction	Manip + bone 2×/wk × 4wk	Cobb↓, rotation↓, FBT↑, 95%efficacy
Li 2021	30/30	13.32 ± 2.61/13.51 ± 2.21	Myofascial massage + Schroth exercises/Schroth exercises	Massage 1×/wk + Schroth 3×/wk ×24wk	Rotation↓, Cobb↓, CBD↓, balance↑
LiLT 2023	38/38	13.07 ± 1.28/13.25 ± 2.03	Chiropractic + 3D traction + brace/3D traction + brace	Chiro 2×/wk + Traction (daily→QOD) + Brace ≥16 h/d × 3mo	84.2% eff, Cobb↓ (19°→8°), lung↑
Lin 2017	36/36	12.1 ± 2.4/12.3 ± 2.2	Bone-setting massage + acupuncture/Brace + traction	Bone-setting 2×/wk + Acup 1×/d (10d)	91.7% eff, Cobb↓ (20.5°→8.6°)
LiWB 2023	24/22	14.04 ± 2.07/13.59 ± 2.84	Lever positioning + 3D bed traction/Conventional lumbar manipulation + 3D traction	Traction QOD + alt. massage ×4mo	91.7% eff, Cobb↓, VAS↓
LiZY 2019	36/36	12.15 ± 0.93/12.12 ± 0.93	Electroacupuncture + massage + apical vertebral rotation/Massage + apical vertebral rotation	EA 30 min + massage 20 min QOD ×6–8wk	88.9% eff, Cobb↓, QoL↑
Luo 2019	37/39	12.68 ± 1.53/12.18 ± 1.59	Home exercises + 3D massage/Home exercises	Massage 3×/wk (30 min) + daily exercise ×12wk	Cobb↓ (3.95° vs 2.36°), back morphology↑
Luo 2021	54/53	13.02 ± 1.46/12.71 ± 1.72	Home spinal exercises + 3D massage/Home spinal exercises	Massage 3×/wk (30 min) + daily exercise ×3mo	Cobb↓, back tilt↓, mental health↑, self-image↑
Mi 2024	25/24	11.32 ± 2.33/11.75 ± 2.42	Shutong spine correction/Bone-setting	3×/wk ×3 courses (12wk)	96% eff, Cobb↓, SRS-22↑(pain/self-image/mental)
Qian 2007	90/30	8.73 ± 0.56/8.60 ± 0.56	Daoyin manipulation/Observation	1×/d (10d): Daoyin 40 min + manip 20 min	Cobb↓, 24.4% cure, 85.6% eff, AE-free
Ren 2021	33/32	15.21 ± 2.12/15.09 ± 2.31	Spine correction + acupuncture/Spine correction	Spine correction + exercise QOD ×8wk	100% eff, Cobb↓, Nash-Moe↓, muscle balance↑
Shen 2016	58/49	9.1 ± 0.44/8.92 ± 0.51	Spinal balance Daoyin + massage + small needle knife/Milwaukee brace	Daoyin 2×/d + Massage 3×/wk + SNK 1×/wk ×12mo	62.5% Cobb correction, lung function↑
Tian 2021	30/30	13.15 ± 2.0/13.35 ± 2.25	Segmented spinal massage + modified Schroth exercises/Modified Schroth exercises	Massage 1×/wk + Exercise 3×/wk ×24wk	Cobb↓ (14.2° vs 17.9°), trunk rotation↓ (5.5° vs 7.2°)
Wang 2014	50/50	10–20/10–20	“Supine traction” + traditional massage/Mechanical traction	1×/d, 5×/wk ×5wk	Early eff↑ (1/3wk), cure↑ (5wk), Cobb↓
Wang 2023	31/31	14.29 ± 2.82/13.29 ± 3.08	Herbal fumigation + routine rehabilitation/Routine rehabilitation	Fumig 1×/d + Rehab 2×/d × 12wk	Rotation↓, Cobb↓, VAS↓, ODI↓, QoL↑
Wang DX 2024	51/51	15.49 ± 3.22/15.52 ± 3.16	TCM acupuncture + routine physiotherapy + core training/Routine physiotherapy + core training	Acup 1×/d + Train 3×/wk ×8wk	Cobb↓, Rotation↓, ODI↓, Adverse↓
Wang JL 2018	41/41	14.09 ± 2.40/14.32 ± 2.45	Electroacupuncture/Electroacupuncture + bone-setting massage	EA 3×/wk (20 min) + Bone-setting 2×/wk ×3mo	90.2% eff, Rotation↓, Cobb↓, VAS↓
Wang X 2019	45/45	17.5 ± 0.5/17.3 ± 0.6	4D traction + tendon-spine manipulation/Conventional traction + tendon-spine manipulation	4D traction 30 min/d + manip 1×/d × 20d	95.6% eff, Cobb↓(21°→8°), >control
Wei 2015	58/49	9.1 ± 0.4/8.9 ± 0.6	Daoyin + massage + acupressure/Brace	Daoyin 2×/d + Massage 2×/wk + Acup 1×/wk ×12mo	Cobb↓, Lung↑, AEMG 1.1, 24mo sustain
Xu 2022	30/30	12.37 ± 1.25/12.57 ± 1.31	"Three-step seven-method” massage/Exercise therapy	1×/d (30 min) × 8wk	93.3% eff, Cobb↓(21.7°→12.5°), SRS-22↑, Strength↑
Yang 2015	42/42	11–23/11–22	Tendon manipulation + bone-setting/Orthotic brace	3 courses ×6wk	95.2% eff, Cobb↓(32°→6°, *Δ*26°), >>control

**Figure 3:**
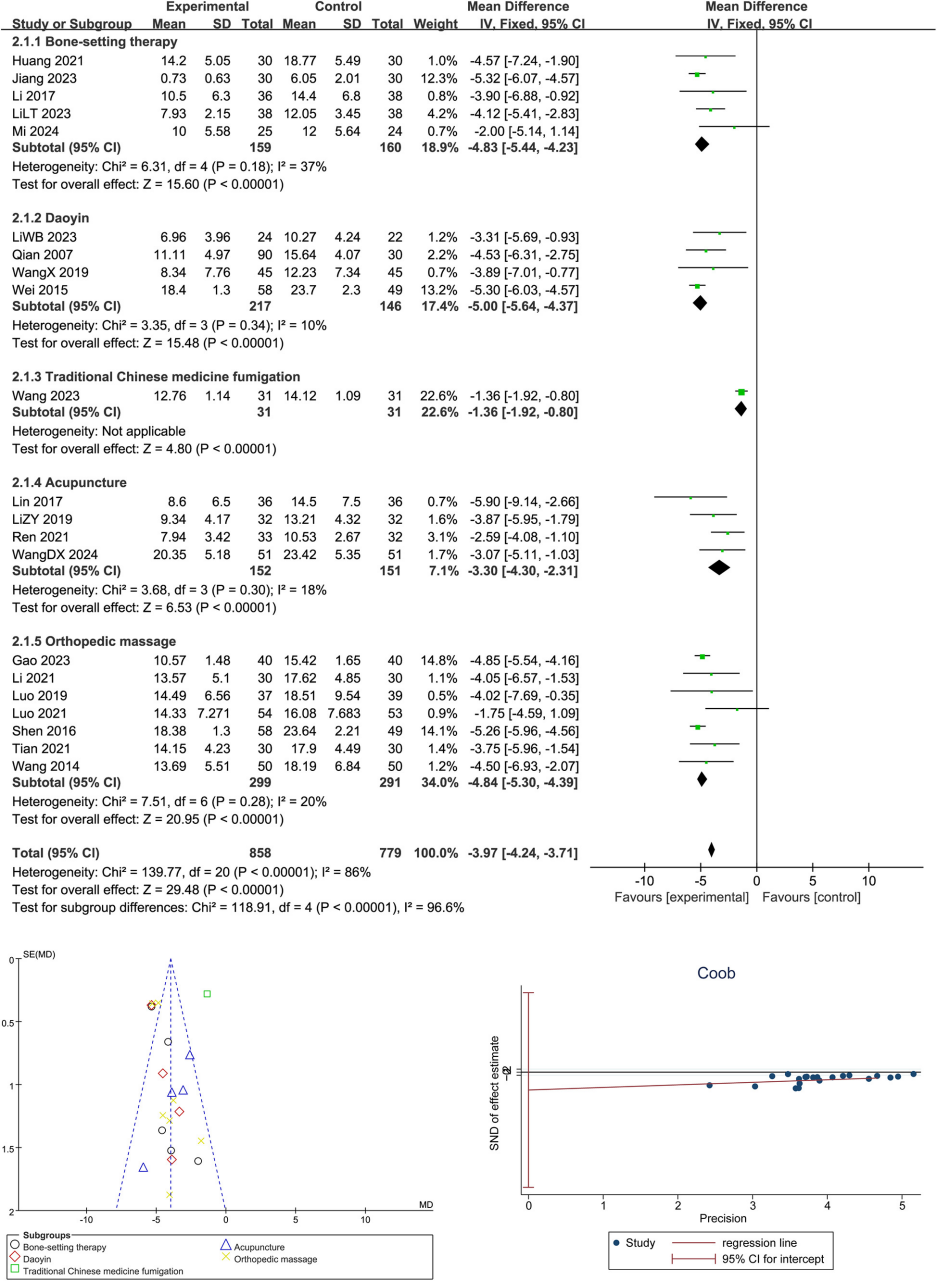
Forest plot, Egger's test, and funnel plot for the effects of Traditional Chinese Medicine on cobb angle in adolescent scoliosis.

As shown in Figure 3, a total of 21 randomized controlled trials involving 858 participants were included to evaluate the effect of Traditional Chinese Medicine (TCM) interventions on Cobb angle in adolescent scoliosis. The overall pooled mean difference significantly favored the TCM group (*Z* = 29.48, *p* < 0.00001), indicating a notable improvement in spinal alignment. Subgroup analysis revealed that all five intervention types were effective: bone-setting therapy (*I*^2^ = 37%, *Z* = 15.60, *p* < 0.00001), Daoyin exercise (*I*^2^ = 10%, *Z* = 15.48, *p* < 0.00001), TCM fumigation (*Z* = 4.80, *p* < 0.00001), acupuncture (*I*^2^ = 18%, *Z* = 6.53, *p* < 0.00001), and orthopedic massage (*I*^2^ = 20%, *Z* = 20.95, *p* < 0.00001). A statistically significant subgroup difference was detected (Chi^2^ = 118.91, df = 4, *p* < 0.00001; *I*^2^ = 96.6%), suggesting that the type of TCM modality influenced treatment outcomes. Egger's regression test showed no significant publication bias, and the funnel plot demonstrated a relatively symmetric distribution, supporting the reliability and robustness of the findings.

**Figure 4:**
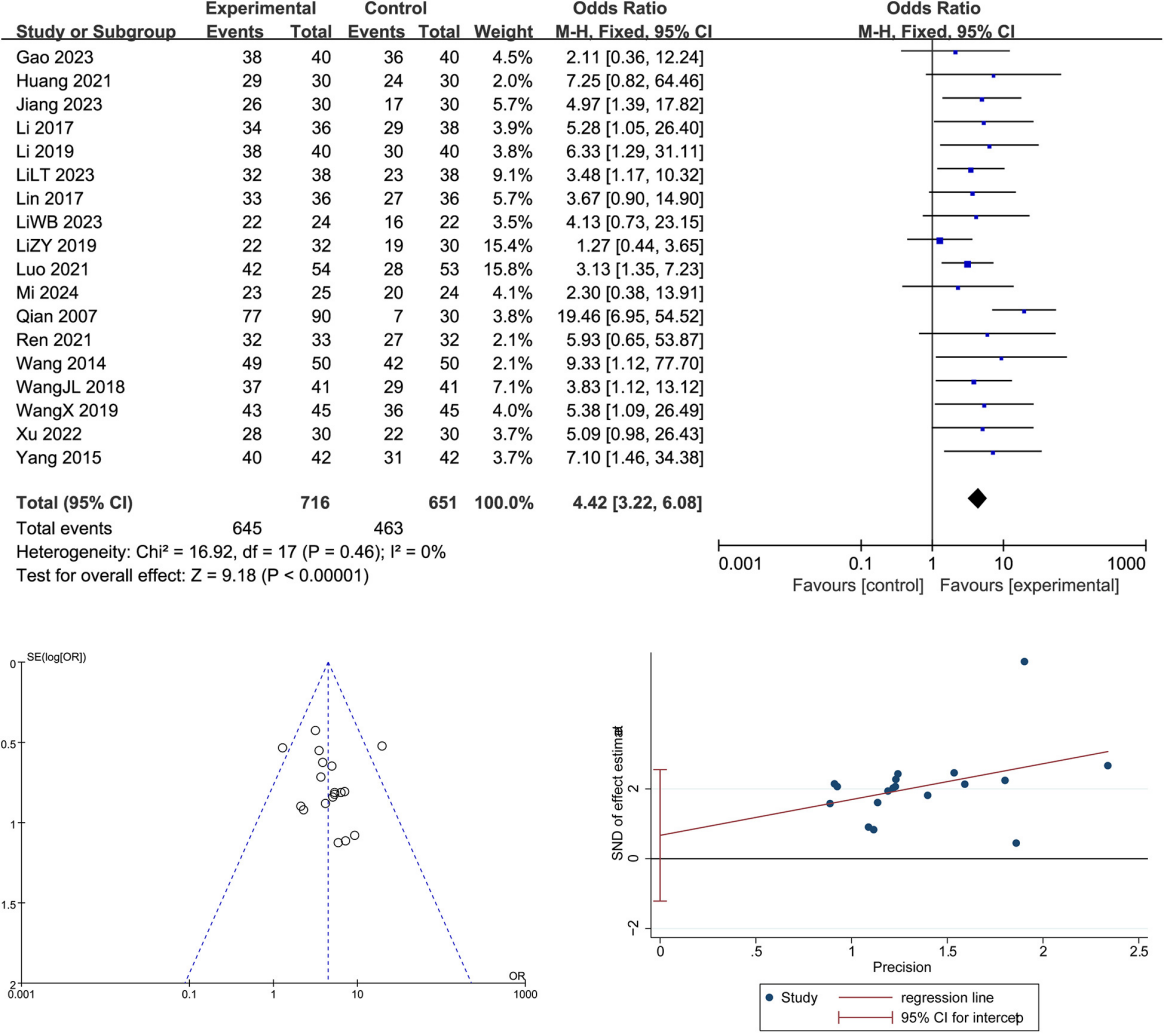
Forest plot, Egger's test, and funnel plot of the overall effectiveness of Traditional Chinese Medicine in adolescent scoliosis.

As shown in Figure 4, a total of 18 randomized controlled trials involving 1,108 participants (645 in the intervention group and 463 in the control group) were included to evaluate the overall effectiveness of Traditional Chinese Medicine (TCM) in treating adolescent scoliosis. The pooled analysis showed a significant improvement in the TCM group compared to controls, with a combined odds ratio of 4.42 [95% CI: 3.22–6.08], indicating that patients receiving TCM were more than four times as likely to achieve clinical effectiveness (*Z* = 9.18, *p* < 0.00001). No significant heterogeneity was observed among the included studies (Chi^2^ = 16.92, df = 17, *p* = 0.46; *I*^2^ = 0%), supporting the stability of the results. Egger's test indicated no significant publication bias, and the funnel plot displayed a symmetrical distribution of studies, suggesting a low risk of small-study effects and confirming the robustness of the evidence.

Overall, traditional Chinese medicine interventions demonstrated significant improvements in adolescent idiopathic scoliosis. Specifically, Cobb angle showed a significant reduction (MD = −3.97°, 95% CI: −4.24 to −3.71, *p* < 0.00001), albeit with substantial heterogeneity (*I*^2^ = 96.6%). In contrast, treatment efficacy showed a robust improvement (OR = 4.42, 95% CI: 3.22–6.08, *p* < 0.00001) with no observed heterogeneity (*I*^2^ = 0%).

## 4 Discussion

### 4.1 Traditional Chinese Medicine demonstrates significant efficacy in reducing cobb angle in AIS patients

This meta-analysis included 21 randomized controlled trials involving 858 adolescents with adolescent idiopathic scoliosis (AIS), and the pooled results showed that Traditional Chinese Medicine (TCM) interventions significantly reduced the Cobb angle, with a combined mean difference of −3.97° (95% CI: −4.24 to −3.71, *p* < 0.00001), supporting the efficacy of TCM as a non-surgical approach for spinal correction. Subgroup analysis revealed that multiple TCM modalities, including bone-setting, Daoyin exercises, massage therapy, acupuncture, and herbal fumigation, all showed statistically significant benefits, with bone-setting (−4.83°) and Daoyin (−5.00°) achieving the greatest reductions. Compared with previous studies, such as (11), which found improvement in pulmonary function through Daoyin and small-needle knife techniques, or (14), who reported Cobb angle improvements via Daoyin and massage, this study advances the field by quantitatively synthesizing outcomes across multiple high-quality RCTs and directly comparing TCM modalities. Unlike (15), who reported only a trend of improvement with chiropractic techniques in case series, our findings offer high-level evidence with defined effect sizes. The mechanisms underlying these effects are likely multifactorial: bone-setting and traction techniques restore vertebral alignment and relieve mechanical constraints; Daoyin exercises improve neuromuscular coordination, enhance postural control, and activate deep trunk stabilizers; acupuncture may modulate spinal reflex arcs and proprioceptive signaling through the central nervous system, while massage therapy improves soft tissue compliance and muscle balance, contributing to spinal symmetry. Moreover, certain TCM interventions may also affect thoracic mobility and respiratory efficiency, indirectly supporting spinal dynamics. Cobb angle assessment tools varied, with some studies using 2D radiographs and others employing 3D imaging, which may introduce inconsistency. Additionally, many studies involved small sample sizes, lacked long-term follow-up, and provided limited data on adherence and protocol fidelity. Future studies should aim to standardize TCM intervention protocols, adopt uniform imaging-based evaluation methods, conduct long-term follow-up, and incorporate objective outcome measures such as muscle activity, balance function, and biomechanical modeling to elucidate underlying mechanisms and enhance the global credibility and applicability of TCM for AIS treatment. To address these limitations, we acknowledge that the observed heterogeneity in Cobb angle results may be attributed to several methodological factors, including the use of different radiographic assessment tools (e.g., 2D vs. 3D imaging), diverse and non-standardized TCM intervention protocols across studies, and small sample sizes in several trials that may reduce the generalizability of findings. Additionally, some studies lacked clear descriptions of treatment frequency and intensity, further contributing to protocol variability. In response, future studies should aim to standardize TCM intervention procedures, adopt uniform imaging-based evaluation methods, and incorporate objective biomechanical outcomes such as electromyographic activity and postural control measurements. Moreover, long-term follow-up should be conducted to evaluate the sustainability of treatment effects and validate the clinical value of TCM over time.

### 4.2 Overall treatment efficacy supports integration of TCM into conservative management

This meta-analysis included 18 randomized controlled trials involving 1,296 adolescents with AIS to evaluate the overall clinical efficacy of Traditional Chinese Medicine (TCM) compared to control interventions. The pooled analysis yielded a statistically significant odds ratio of 4.42 (95% CI: 3.22–6.08, *p* < 0.00001), indicating that patients receiving TCM interventions were over four times more likely to achieve clinical improvement than those in the control group, with no observed heterogeneity (*I*^2^ = 0%). These findings demonstrate that TCM not only reduces the Cobb angle but also contributes meaningfully to broader therapeutic goals, including symptom relief, functional improvement, and quality of life enhancement. Compared with previous high-level studies such as (16), which confirmed the efficacy of bracing in halting scoliosis progression but raised concerns about psychological discomfort and compliance issues, TCM provides a more holistic and patient-centered alternative. Likewise (17, 18), emphasized the need for integrating conservative strategies with a biopsychosocial framework; our findings support this view by presenting TCM as a valuable adjunct that addresses both structural and symptomatic dimensions of AIS. Mechanistically, the comprehensive benefits of TCM can be attributed to its multi-target approach: bone-setting and massage techniques restore musculoskeletal balance and joint mobility; acupuncture modulates pain pathways and neuromuscular coordination; Daoyin exercises enhance core stability and spinal proprioception; and herbal fumigation promotes circulation and reduces inflammation. The combination of these modalities likely produces synergistic effects, reinforcing physical correction with physiological and psychosocial recovery. Moreover, improvements in patient-reported outcomes such as pain reduction (e.g., VAS scores), mental well-being, and self-image observed in several included trials further validate the systemic nature of TCM interventions. However, this study also has limitations. Although the heterogeneity was low for overall efficacy, variation still existed in treatment protocols, outcome definitions, and follow-up durations. Many included studies lacked blinding, which may overestimate treatment effects, and the criteria used to define “effective treatment” were not standardized across all trials. Additionally, while short-term improvements were evident, long-term outcomes remain underreported, raising concerns about the durability of treatment benefits. Future research should aim to develop standardized clinical pathways that integrate TCM with conventional rehabilitation protocols, include long-term follow-up, and incorporate multidimensional outcome measures such as biomechanical function, mental health, and social participation to fully elucidate the role of TCM in the conservative management of AIS. Although the overall treatment efficacy analysis demonstrated no significant heterogeneity, methodological inconsistencies were still present, particularly in the definition of “clinical effectiveness,” follow-up durations, and study design features. Many trials lacked blinding procedures, which may introduce performance or detection bias and inflate reported effect sizes. Furthermore, the short follow-up periods used in most studies limit our ability to determine the durability of treatment outcomes. To enhance clinical translation, future research should develop standardized clinical pathways integrating TCM into multidisciplinary conservative management strategies for AIS, establish consensus on treatment efficacy criteria, and assess long-term outcomes using multidimensional measures such as biomechanical function, psychological status, and health-related quality of life.

## 5 Conclusion

This meta-analysis demonstrates that Traditional Chinese Medicine (TCM) interventions—especially spinal manipulation, Daoyin exercises, acupuncture, and massage—are effective in improving both structural and clinical outcomes in adolescents with idiopathic scoliosis. The findings highlight clear clinical applications: TCM can be implemented as a structured, non-invasive regimen combining manual therapy (2–3 sessions/week), daily posture-corrective Daoyin, and supportive acupuncture and massage over a 12-week period, offering a patient-friendly alternative to bracing or surgery. To enhance clinical adoption, future studies should develop standardized TCM treatment protocols, evaluate cost-effectiveness, and establish guidelines for integration into routine scoliosis rehabilitation. Moreover, future research should be more specific in targeting long-term outcomes, biomechanical mechanisms, and comparative efficacy across different scoliosis subtypes and severity levels through multicenter randomized trials.

## 6 Limitations

This study has several limitations. First, variations in TCM intervention types, treatment frequency, and outcome measures across studies may have contributed to heterogeneity. Second, some trials lacked clear descriptions of randomization and blinding, increasing the risk of bias. Third, most studies had small sample sizes, short intervention periods, and limited follow-up, restricting generalizability. Lastly, inconsistent criteria for defining treatment effectiveness affected the comparability of results. More high-quality, large-scale RCTs are needed to validate these findings.

## Data Availability

The datasets presented in this study can be found in online repositories. The names of the repository/repositories and accession number(s) can be found in the article/Supplementary Material.
